# Sul-BertGRU: an ensemble deep learning method integrating information entropy-enhanced BERT and directional multi-GRU for S-sulfhydration sites prediction

**DOI:** 10.1093/bioinformatics/btaf078

**Published:** 2025-02-20

**Authors:** Xirun Wei, Qiao Ning, Kuiyang Che, Zhaowei Liu, Hui Li, Shikai Guo

**Affiliations:** Department of Information Science and Technology, Dalian Maritime University, Dalian 116026, P.R. China; Department of Information Science and Technology, Dalian Maritime University, Dalian 116026, P.R. China; School of Artificial Intelligence and Computer Science, Jiangnan University, Wuxi 214122, P.R. China; Key Laboratory of Symbolic Computation and Knowledge Engineering of Ministry of Education, Jilin University, Changchun 130012, P.R. China; Neutech Group Limited, Dalian 116023, P.R. China; Department of Information Science and Technology, Dalian Maritime University, Dalian 116026, P.R. China; Department of Information Science and Technology, Dalian Maritime University, Dalian 116026, P.R. China; Department of Information Science and Technology, Dalian Maritime University, Dalian 116026, P.R. China; Department of Information Science and Technology, Dalian Maritime University, Dalian 116026, P.R. China; The Dalian Key Laboratory of Artificial Intelligence, Dalian 116026, P.R. China

## Abstract

**Motivation:**

S-sulfhydration, a crucial post-translational protein modification, is pivotal in cellular recognition, signaling processes, and the development and progression of cardiovascular and neurological disorders, so identifying S-sulfhydration sites is crucial for studies in cell biology. Deep learning shows high efficiency and accuracy in identifying protein sites compared to traditional methods that often lack sensitivity and specificity in accurately locating nonsulfhydration sites. Therefore, we employ deep learning methods to tackle the challenge of pinpointing S-sulfhydration sites.

**Results:**

In this work, we introduce a deep learning approach called Sul-BertGRU, designed specifically for predicting S-sulfhydration sites in proteins, which integrates multi-directional gated recurrent unit (GRU) and BERT. First, Sul-BertGRU proposes an information entropy-enhanced BERT (IE-BERT) to preprocess protein sequences and extract initial features. Subsequently, confidence learning is employed to eliminate potential S-sulfhydration samples from the nonsulfhydration samples and select reliable negative samples. Then, considering the directional nature of the modification process, protein sequences are categorized into left, right, and full sequences centered on cysteines. We build a multi-directional GRU to enhance the extraction of directional sequence features and model the details of the enzymatic reaction involved in S-sulfhydration. Ultimately, we apply a parallel multi-head self-attention mechanism alongside a convolutional neural network to deeply analyze sequence features that might be missed at a local level. Sul-BertGRU achieves sensitivity, specificity, precision, accuracy, Matthews correlation coefficient, and area under the curve scores of 85.82%, 68.24%, 74.80%, 77.44%, 55.13%, and 77.03%, respectively. Sul-BertGRU demonstrates exceptional performance and proves to be a reliable method for predicting protein S-sulfhydration sites.

**Availability and implementation:**

The source code and data are available at https://github.com/Severus0902/Sul-BertGRU/.

## 1 Introduction

Post-translational modifications (PTMs) are vital for regulating cellular activities, including gene transcription, DNA repair, and protein interactions (Ning and Li 2022). Cysteine, a rare amino acid, undergoes various PTMs, particularly through its thiol group, which acts as a molecular switch in processes like redox balance and signaling (Esposito *et al.* 2004) and is linked to diseases like cancer and neurodegenerative disorders (Shi and Carroll 2020, Gupta *et al.* 2022). Among these, S-sulfhydration, though critical in cardiovascular and neurological disorders, is still poorly understood, presenting significant analytical challenges (Mir *et al.* 2014).

Various traditional techniques have been employed to identify S-sulfhydration sites, such as the modified biotin conversion method (Mustafa *et al.* 2009), the maleimide fluorescence method (Sen *et al.* 2012), and selective labeling assays (Zhang *et al.* 2014). While these methods can be precise, they often rely on chemical reagents and suffer from issues like lack of specificity and sensitivity. Additionally, reactions involving maleimides can result in extensive labeling, which may negatively impact the results (Zhang *et al.* 2019).

With the advancement of deep learning, researchers are increasingly using these techniques for predicting protein sites. For instance, Zhang *et al.* (2023) developed BiGRUD-SA to predict S-sulfenylation sites using multiple encodings, BiGRU, self-attention, and deep neural networks (DNN). Zhao *et al.* (2022) introduced Mul-SNO, leveraging BERT preprocessing for S-sulfenylation prediction. Ning *et al.* (2021) proposed GPS-Palm for efficient palmitoylation site prediction using parallel convolutional neural networks (CNNs). Other techniques, such as random forests (Hasan *et al.* 2017) and support vector machines (Kumari *et al.* 2018), are also used for protein site prediction. However, research on predicting S-sulfhydration sites is less developed compared to S-sulfenylation, S-nitrosylation, and palmitoylation. Although Li *et al.*’s pCysMod model (Li *et al.* 2021) predicts S-sulfhydration sites, its performance still falls short for real-world applications. Additionally, current methods do not account for sequence orientation during PTM, leading to potential losses in prediction accuracy.

**Figure 1.**
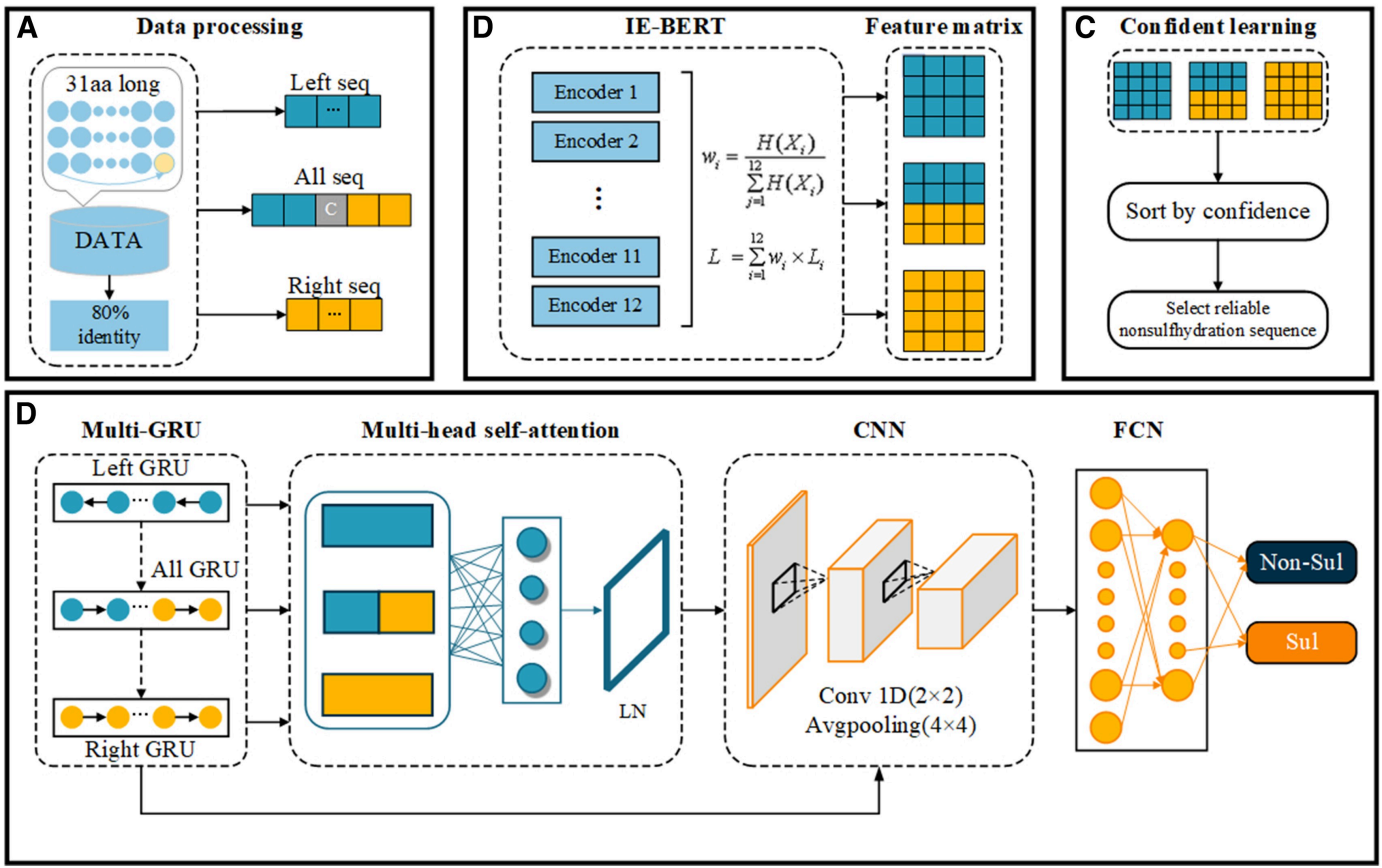
Schematic view of the Sul-BertGRU architecture. (A) Data processing module divides the protein sequence into left and right sub-sequences. (B) IE-BERT module extracts the initial features of the sequence. (C) Confidence learning module removes noisy data from negative samples. (D) Directional feature extraction module extracts directional and local features from the sequence.

In this work, we propose a new framework for S-sulfhydration sites prediction that integrates multi-directional GRU and an information entropy-enhanced BERT framework (Sul-BertGRU), whose architecture is shown in Fig. 1. Sul-BertGRU framework consists of four modules: data processing module, IE-BERT module, confidence learning module, and directional feature extraction module. Data processing module divides the protein sequence into left and right sub-sequences. IE-BERT module extracts the initial features of the sequence. Confidence learning module removes noisy data from negative samples. Directional feature extraction module extracts directional and local features from the sequence. The principal contributions of this research are as follows:

- We introduce the IE-BERT aggregation technique, which uses informational entropy to enhance prediction efficacy and accuracy by consolidating information across 12 encoder layers in BERT.
- We develop a multi-directional GRU algorithm to extract directional features from different sequence fragments, improving the capture of S-sulfhydration modifications.
- We have proposed a new multi-module deep learning framework for predicting S-sulfhydration sites, which significantly outperforms existing methods.

## 2 Materials and methods

### 2.1 Benchmark datasets

In this work, the dataset is obtained from the iCysMod database (website http://icysmod.omicsbio.info/), identifying 2705 S-sulfhydration sites in proteins. Based on the comparative analysis of different window sizes shown in Supplementary Fig. S6, each site is centered with a 31-amino-acid window (−15 C +15). Peptides with S-sulfhydration are labeled as positive, while those without are labeled as negative. The dataset comprises 2705 positive and 16 697 negative samples. We split 20% of the proteins into an independent test set, while the remaining 80% are divided into training and validation sets. The training set is used for model training, and the validation set helps verify and enhance the model’s performance and generalization.

### 2.2 Protein sequence segmentation

Considering that S-sulfhydration is an enzymatic reaction and that the enzymatic reaction may be directional, we partition the protein sequence according to different directions to explore and extract the positional features and interaction information of the modification sites. Drawing inspiration from previous research (Ning and Sheng 2021), we split the protein sequence into two halves centered on the S-sulfhydration site. Detailed operations are shown in Supplementary Materials.

### 2.3 IE-BERT module for initial feature extraction

Following the division of the protein sequence into left and right halves, we analyze and extract features from the entire sequence as well as from each half. This approach is influenced by the methodology described by Le *et al.* (2021), we propose an information entropy-enhanced BERT model, named IE-BERT, for capturing the key features of protein sequences more adequately.

#### 2.3.1 Preliminary feature extraction based on BERT

Protein sequences are lengthy and complex, making traditional feature extraction methods insufficient. To capture critical information, we employ the BERT model, which effectively handles long-term dependencies in natural language processing (NLP) (Devlin *et al.* 2019). We treat protein sequences as sentences, converting amino acid information into fixed-length feature vectors. By inserting spaces between amino acids, the sequence is reformatted for BERT to analyze as natural language text.

BERT’s feature extraction comprises 12 identical transformer encoder modules, each with two sub-layers (Vaswani *et al.* 2017). The first sub-layer uses multi-head attention to calculate self-attention, while the second is a feed-forward neural network based on residual networks. The output is calculated as:


(1) Output=LayerNorm(x+Sub_layer(x)),

where *x* is the input feature vector, + denotes the residual connection, and *LayerNorm* applies normalization.

In the feed-forward network, each position’s contextual representation undergoes a nonlinear transformation, defined by:


(2) $$\text{FFN}(x)=\text{max}(0,xW_{1}+b_{1})W_{2}+b_{2},$$

where *x* is the feature vector from the attention layer, and $W_{1},\;W_{2},\;b_{1}$ and $b_{2}$ are the weight matrices and bias terms.

BERT is trained in two stages: pre-training on a large corpus and fine-tuning on specific tasks. We follow Le’s approach (Ning and Sheng 2021) using a masked language model method. During training, 15% of tokens are masked, with 80% replaced by [MASK], 10% unchanged, and 10% replaced by random words. Each amino acid residue is ultimately transformed into a 768-dimensional word embedding vector.

#### 2.3.2 Information entropy-based feature aggregation

To mitigate the potential loss of essential features through the numerous nonlinear transformations in the multi-layer encoder, we introduce an aggregation layer after the 12-layer encoder. This layer computes the information entropy for each encoder layer and aggregates the information accordingly.

The information content $I(X_{i})$ in each encoder layer is calculated as:


(3) $$I(X_{i})=-{\text{log}\;}_{2}p(X_{i}),i=1,2,\ldots ,12,$$

where *X_i_* represents the output feature vector, and $p(X_{i})$ is the probability of each category in the encoder layer.

The information entropy $H(X_{i})$ for each encoder layer is given by:


(4) $$H(X_{i})=-\sum_{i=1}^{12}p(X_{i})\;\text{log}\;p(X_{i}).$$

Next, the weight *w_i_* of each layer is computed based on its information entropy:


(5) $$w_{i}=\frac{H(X_{i})}{\sum_{i=1}^{12}H(X_{i})}.$$

Finally, the weighted sum *L* is calculated as:


(6) $$L_{}=\sum_{i=1}^{12}w_{i}\times L_{i}$$

where $L_{i}$ represents the corresponding layer value.

### 2.4 Confident learning for deleting mislabeled samples

Due to limitations in biotechnology, biological experiments can identify S-sulfhydration sites but not nonsulfhydration sites, leading to potential mislabeled samples in the negative dataset. To minimize the impact of these mislabeled samples on model training, we apply confident learning to identify and eliminate them.

Confident learning, a method proposed by researchers at MIT and Google (Northcutt *et al.* 2021), includes five techniques to filter incorrect samples. We use the first method, *C*_confusion_, to filter negative samples. The process begins with cross-validation of the Sul-BertGRU model on the training set, where we calculate the probability P[i][j] of sample *i* belonging to category *j*. We then compute the average probability for each manually calibrated category *j*, as a confidence threshold, t[j], using the following formula:


(7) $$t_{j}=\frac{1}{\left|N_{\tilde{y}=j}\right|}\sum_{n\in N_{\tilde{y}=j}}\hat{p}(\tilde{y}=j;n,\theta ).$$

Here, $\tilde{y}$ is the noise label, *n* is a sample in the dataset, θ are the model parameters, $N_{\tilde{y}=j}$ is the subset labeled as *j*, and $\hat{p}(\tilde{y}=j;x,\theta )$ represents the predicted probability of classifying as *j*.

Samples with a maximum prediction probability P[i][j] lower than the threshold t[j] are considered error samples. After eliminating these erroneous samples, the final nonsulfhydration sequence feature vector $L_{t}$ is obtained and fed into the directional feature extraction module for further processing.

### 2.5 Directional feature extraction module

GRU module, multi-head self-attention mechanisms, CNN module, and fully convolutional networks (FCN) module are used to create a framework for further extracting directional features and identifying S-sulfhydration sites. The components of this framework are elaborated in the subsequent subsections.

#### 2.5.1 Multi-GRU module

Recurrent neural networks (RNNs) are widely used to capture temporal sequence information in various fields, including state of charge estimation for lithium-ion batteries (Chen *et al.* 2023) and leukemia prediction (Abbasi *et al.* 2024). However, traditional RNNs face the “gradient vanishing” problem with long protein sequences, limiting their ability to capture long-term dependencies (Ding *et al.* 2018). To address this, we use GRU, which, similar to long short-term memory (LSTM), has fewer parameters. GRU merges the input and forget gates into an update gate and consolidates the memory unit and hidden layer into a reset gate, effectively solving the “gradient vanishing” issue (Chung *et al.* 2014). Our multi-GRU module uses three GRU models to capture directional features of the sequence’s left half, right half, and entirety.

The update gate *z_t_* for timestep *t* is computed as:


(8) $$z_{t}=\sigma (W^{\left(z\right)}L_{t}+U^{\left(z\right)}M_{t-1}),$$

where *L_t_* is the feature vector from BERT, and $M_{t-1}$ is the previous timestep’s data. The reset gate *r_t_* is similarly calculated:


(9) $$r_{t}=\sigma (W^{\left(r\right)}L_{t}+U^{\left(r\right)}M_{t-1}).$$

Next, the new memory content ${\tilde{M}}_{t}$ is created as:


(10) $${\tilde{M}}_{t}=\tanh(W^{\left(m\right)}L_{t}+r_{t}⊙U^{\left(m\right)}M_{t-1}).$$

Finally, the memory $M_{t}$ for the current timestep is computed as:


(11) $$M_{t}=z_{t}⊙M_{t-1}+(1-z_{t})⊙{\tilde{M}}_{t}.$$

The feature matrix $M_{t}$ is then used as input for the multi-head attention mechanism and the CNN module.

Considering the directionality of enzymatic reactions, the three GRU models process protein sequences in various orientations (e.g. LLL, LRL, LLR, etc.).

#### 2.5.2 Multi-head self-attention module

To capture long-term dependencies in protein sequences, we adopt the self-attention mechanism, widely recognized for its robust capability in various tasks such as visual recognition (Li *et al.* 2023), image classification (Zhang *et al.* 2024a), and disease prediction (Zhang *et al.* 2024b). The feature information of the three parts of the protein sequences is extracted, and further processed with a multi-head self-attention module to focus on the important information, generating feature matrix *A*. Detailed processes are in Supplementary Materials.

#### 2.5.3 CNN module

To address the concern of GRU and self-attention mechanisms for sequence-wide dependencies, which may overlook local details, we input their feature matrices into a CNN, consisting of three convolutional and pooling layers, captures and fuses richer local features between amino acid residues. Follow the process in Supplementary Materials, we generate the ultimate feature matrix *Y*, which is then input into a fully connected network for subsequent classification and prediction tasks.

### 2.6 Classification and prediction module

To leverage the features extracted by Sul-BertGRU for binary classification, we design a prediction module consisting of an FCN with two fully connected layers. The FCN converts the multidimensional features from the directional extraction module into feature vectors, applying a sigmoid activation function to output class probabilities. Dropout regularization at a 0.5 rate is used to enhance model generalization.

## 3 Results and experiments

### 3.1 Ablation experiments

The directional feature extraction module includes multi-GRU, multi-head self-attention, and CNN components. We assess multi-GRU’s efficacy by replacing it with GRU and CNN and compare these configurations. Additionally, we evaluate the impact of removing the CNN and multi-head self-attention modules individually.

As shown in Table 1, the multi-GRU module outperforms other configurations. Removing CNN results in at least a 3% decrease in all metrics. Although GRU performs slightly better in *Sn* and *Sp*, it shows a significant drop in other metrics. This demonstrates the superiority of the multi-GRU framework for classification. Removing the multi-head self-attention module results in decreases of 6.16% in *Sn*, 1.46% in *Acc*, 1.85% in *MCC*, and 1.23% in *AUC*, highlighting its role in focusing on critical features. The removal of the CNN module decreased *Sp*, *Pre*, *Acc*, *MCC*, and *AUC* decrease by 4.72%, 2.61%, 2.05%, 3.84%, and 2.17%, respectively, underscoring its importance in localized feature extraction.

**Table 1.** Results of ablation experiments for five frameworks.^a^

Framework	Sn	Sp	PRE	ACC	MCC	AUC
CNN	0.6978	0.6557	0.6900	0.6777	0.3537	0.6767
GRU	0.7295	**0.7357**	0.7519	0.7324	0.4647	0.7326
GRU-ATT	**0.8619**	0.6352	0.7219	0.7539	0.5129	0.7486
GRU-CNN	0.7966	0.7193	**0.7571**	0.7598	0.5180	0.7580
Multi-GRU	0.8582	0.6824	0.7480	**0.7744**	**0.5513**	**0.7703**

a The bold font indicates the maximum value of each metric and the underlined font indicates the second largest value of each metric.

### 3.2 Hyperparameters analysis

Hyperparameters significantly affect the model’s predictive capabilities. Thus, we utilize 10-fold cross-validation to examine how certain parameters influence prediction outcomes and to assess and compare the metrics’ values.

#### 3.2.1 The hyperparameters in GRU

**Figure 2.**
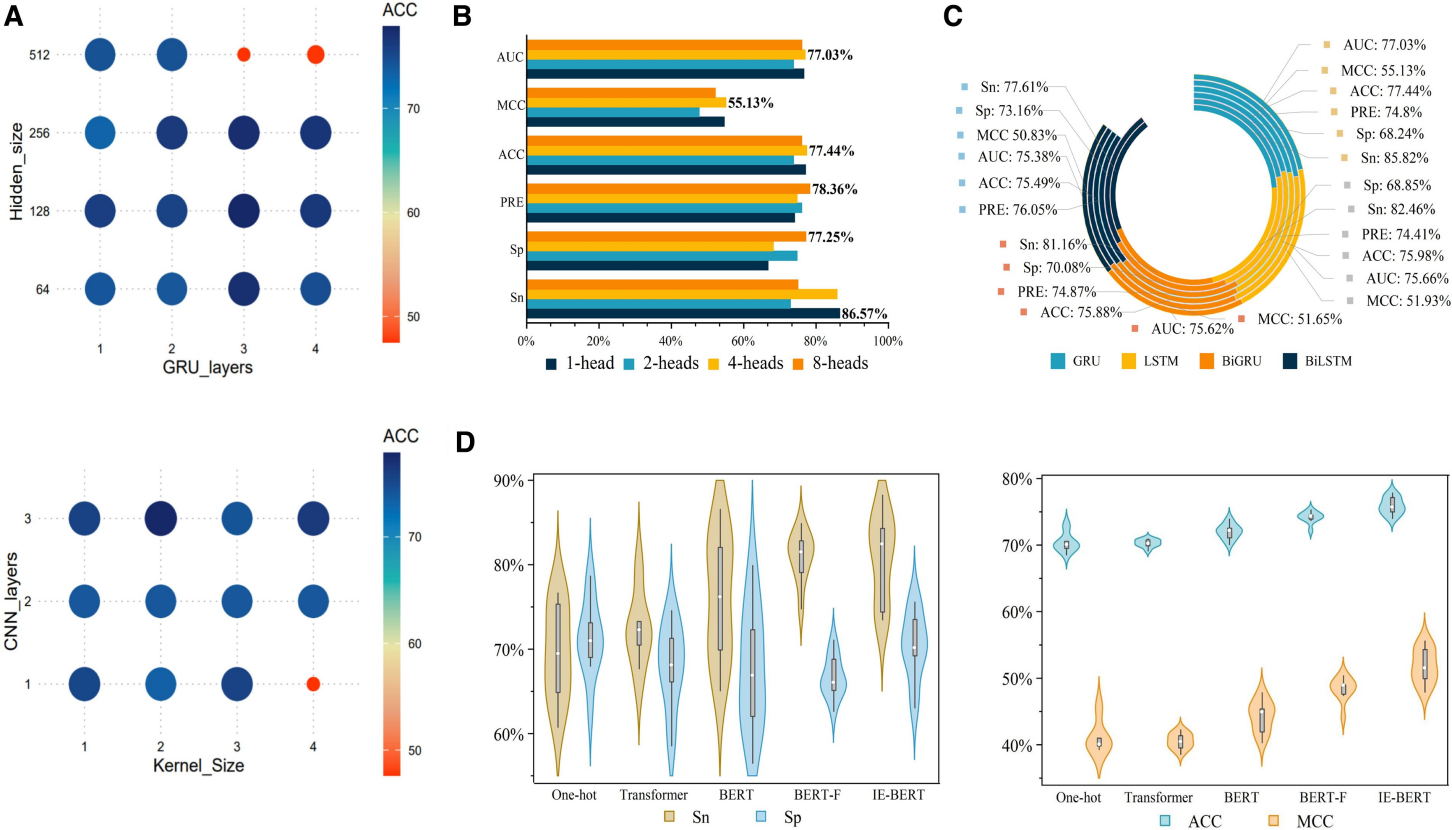
(A) Performance comparison of GRU and GCN hyperparameters on prediction Acc (the color of point) and MCC (the size of point). (B) Performance with different heads of multi-head self-attention module. (C) Performance comparison of four models for extracting directional features. (D) Performance comparison between five methods for extracting features.

For the GRU model, increasing the hidden layer dimensions and the number of layers is crucial for improving the model’s capacity to learn richer and more complex features, enabling better handling of long protein sequences. In this study, we experiment with hidden layer dimensions of 64, 128, 256, and 512, and layer numbers of 1, 2, 3, and 4. We evaluate the model’s predictive performance using *Acc* and *MCC* as metrics, as shown in Fig. 2A. The optimal configuration, with three GRU hidden layers and a dimension of 128, yielded the best results, effectively identifying S-sulfhydration sites. Detailed analysis is provided in the Supplementary Materials.

#### 3.2.2 The hyperparameters in CNN

In CNN, the performance is significantly influenced by the convolutional kernel size and the number of convolutional layers. We conduct a series of validation experiments to explore these factors, setting kernel sizes to 1, 2, 3, and 4, and the number of CNN layers to 1, 2, and 3. *Acc* and *MCC* are used as evaluation metrics, as shown in Fig. 2A. The optimal configuration—yielding the highest *Acc* and *MCC*—is achieved with a kernel size of 2 and 3 CNN layers. Detailed experiments and analysis are provided in the Supplementary Materials.

#### 3.2.3 Number of heads in multi-head self-attention

For the multi-head self-attention mechanism, selecting the optimal number of attention heads is crucial for effective feature extraction. We conduct experiments with 1, 2, 4, and 8 attention heads to explore their impact, as shown in Fig. 2B. The optimal performance is achieved with 4 attention heads, as it provide the best balance between feature extraction and model complexity. Detailed experiments and analysis are provided in the Supplementary Materials.

### 3.3 Impact of GRU model

In Sul-BertGRU, we use GRU to extract directional features of protein sequences and compare its performance with LSTM and bidirectional models (BiGRU and BiLSTM), as shown in Fig. 2C. GRU performs best among the models, with improvements of 1.31% in *Sn*, 2.46% in *Sp*, 1.82% in *Pre*, 3.72% in *Acc*, and 1.88% in *MCC* over LSTM. This advantage may be due to GRU’s fewer parameters, which reduces overfitting. While BiLSTM slightly outperforms GRU, it shows reduced performance in other metrics. The comparison indicates that bidirectional models, BiGRU and BiLSTM, have varying performances when considering both upstream and downstream sequence information. Detailed results are provided in the Supplementary Materials.

### 3.4 Effectiveness of information entropy-enhanced BERT

In extracting protein sequence features, textual representations are converted into vectors or matrices, which are categorized into discrete and distributed representations. Discrete representation uses One-Hot encoding, which converts categorical labels into binary vectors reflecting positional relationships among amino acids (Rodríguez *et al.* 2018). Distributed representation, or word embedding, maps high-dimensional word vectors to a lower-dimensional space, capturing conceptual essence. The Transformer model (Northcutt *et al.* 2021) uses stacked encoders and decoders. BERT, based on the Transformer, uses only the encoder with bidirectional encoding, improving semantic extraction. BERT-F enhances BERT by aggregating outputs from all 12 encoder layers. Our proposed IE-BERT model improves on this by using information entropy to weight outputs from each encoder layer. To ensure the rigor of the experiments, we conducted 10 repetitions and reported the results for metrics.

Comparing these methods, IE-BERT outperforms One-Hot, Transformer, and BERT in *Sn*, *Sp*, *Pre*, *Acc*, *MCC*, and *AUC*, as shown in Fig. 2D. One-Hot captures amino acid distinctions but misses correlations and order. Transformers struggle with complex protein sequences, while BERT’s two-stage training improves performance. BERT-F aggregates features from all encoder layers, and IE-BERT further enhances this by weighting layer outputs based on information entropy, improving prediction and generalization.

### 3.5 Performance comparison of different protein sequence directions

Protein PTMs often occur in a specific sequence order, suggesting that the formation of S-sulfhydration sites might follow a sequential pattern. We compared eight orientations—LLL, LRL, LLR, LRR, RRR, RLR, RRL, and RLL. The specific details of these eight orientations are as follows:

1. Both halves and the full sequence are processed from left to right (LLL).
2. Left half from left to right, right half from right to left, full sequence from left to right (LRL).
3. Both halves from left to right, with the full sequence from right to left (LLR).
4. Left half from left to right, right half from right to left, and the full sequence also from right to left (LRR).
5. All sequences, including both halves and full, from right to left (RRR).
6. Left half from right to left, right half from left to right, and the full sequence from right to left (RLR).
7. Left half and right half from right to left, with the full sequence from left to right (RRL).
8. Left half from right to left, right half from left to right, and the full sequence from left to right (RLL). After extracting directional features from the three parts of the protein sequence, we input these features into the attention module for processing. Following this, the outputs from both the GRU and attention modules are extracted independently, concatenated, and then input into the CNN module. This process allows for a thorough capture of local features that might be missed otherwise. The results are shown in Table 2.

**Table 2.** Performance results of different protein sequence directions.^a^

Direction	Sn	Sp	PRE	ACC	MCC	AUC
LLL	0.7407	**0.7193**	0.7434	0.7305	0.4599	0.7300
LRL	0.8321	0.6168	0.7046	0.7295	0.4615	0.7244
LLR	**0.8638**	0.6455	0.7280	0.7598	0.5243	0.7546
LRR	0.7892	0.7090	0.7467	0.7510	0.5003	0.7490
RRR	0.8190	0.6844	0.7403	0.7549	0.5093	0.7517
RLR	0.8433	0.6803	0.7434	0.7656	0.5325	0.7618
RRL	0.8358	0.6721	0.7368	0.7578	0.5166	0.7540
RLL	0.8582	0.6824	**0.7480**	**0.7744**	**0.5513**	**0.7703**

a The bold font indicates the maximum value of each metric and the underlined font indicates the second largest value of each metric.

As indicated in Table 2, RLL yielded the best results for *Sn*, *Pre*, *Acc*, *MCC*, and *AUC*, suggesting superior prediction performance. This improvement is due to centering the protein sequence around the cysteine site, which is surrounded by critical features. Extracting features from right to left better captures detailed information about the S-sulfhydration site, as the segment closer to the cysteine gathers more relevant details. Conversely, left-to-right extraction might miss crucial information. Similarly, right-to-left processing of the right half captures more feature information about S-sulfhydration sites.

**Figure 3.**
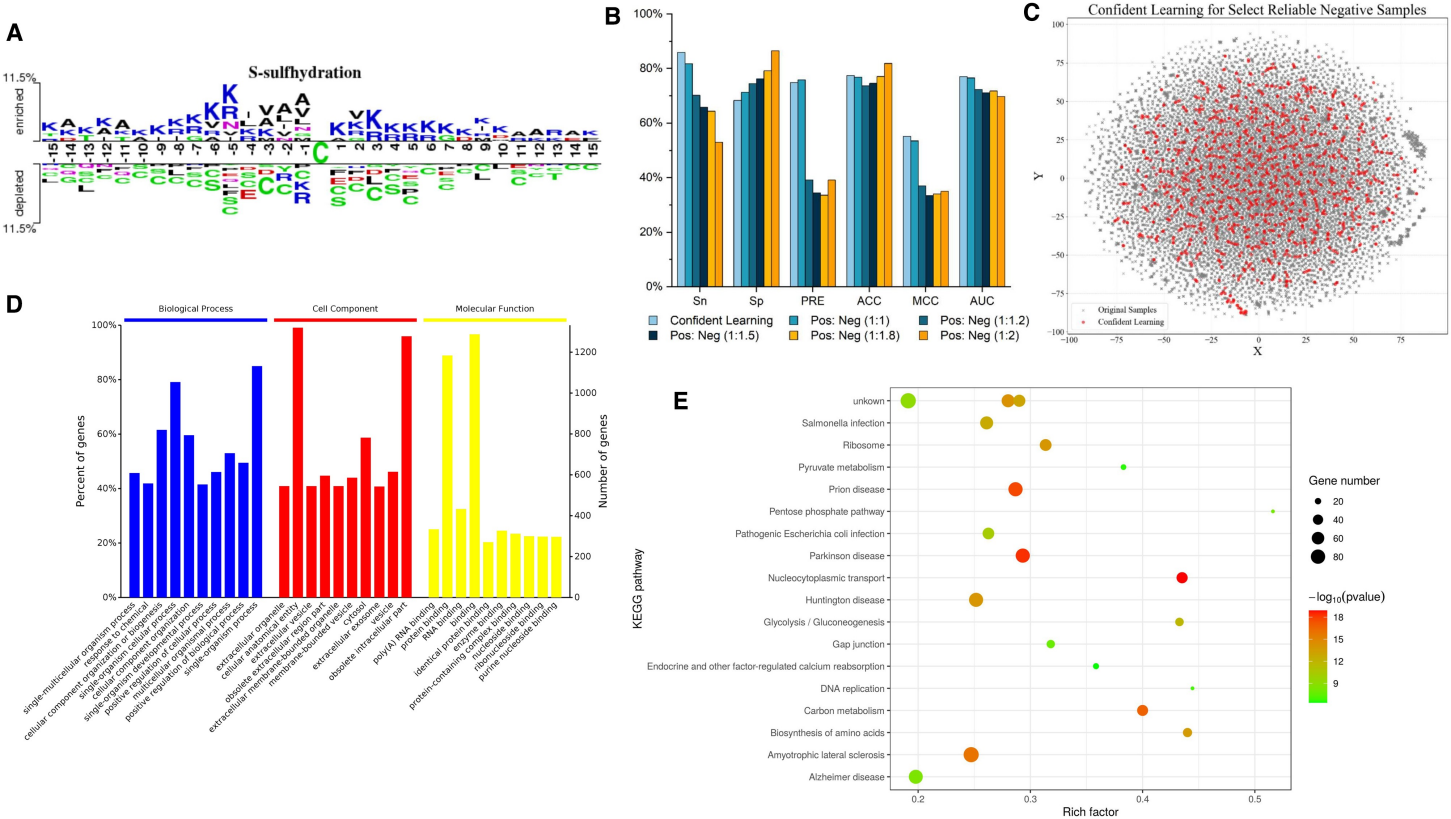
(A) Two sample logos of the compositional biases around S-sulfhydration sites compared with nonsulfhydration sites. (B) Performance comparison using confident learning and random selection of negative samples. (C) t-SNE result of confident learning for selecting reliable negative samples. (D) The top 10 statistically over-represented terms of biological processes, cell component and molecular functions (P-value<.01) (E) Top enriched pathways related to S-sulfhydration modified proteins.

To further assess the effectiveness of different orientations, we also analyzed the specificity of S-sulfhydration and nonsulfhydration sites. Figure 3A shows the sequence conservation patterns, revealing that alanine, lysine, arginine, and valine are more abundant near S-sulfhydration sites, while cysteine, arginine, and serine are more common near nonsulfhydration sites. Overall, there is a decreasing trend in enrichment from S-sulfhydration sites toward both ends of the sequences. Thus, using the RLL direction enhances the model’s classification and prediction performance.

Additionally, to eliminate the influence of biological methods such as mass spectrometry (MS) on the results, we excluded K/R residues and selected sites with only other modifications to compare them with positive samples. After analysis, we found that even when other samples were selected, these sites still exhibited a decreasing trend from the central sulfur atom toward the edges, consistent with our original conclusion. The results are shown in Supplementary Fig. S5.

### 3.6 Analysis of confident learning

To predict S-sulfhydration sites accurately, it’s crucial to address mislabeled samples. We evaluated confidence learning for identifying and improving mislabeled samples by comparing three methods on the test set: (i) applying confidence learning to negative samples to select reliable ones, (ii) selecting a random number of samples equal to the number of positive samples, and (iii) selecting 1.2, 1.5, 1.8, and 2 times the number of positive samples from negatives for training. The results are shown in Fig. 3B.

The model performs best when applying confidence learning to negative samples, showing superior results in *Sn*, *MCC*, and *AUC*. This suggests that mislabeling in nonsulfhydration sequences (actually S-sulfhydration) affects prediction accuracy. Removing these mislabeled samples enhances performance. Confidence learning on all samples significantly improves accuracy, while random selection methods may include potential S-sulfhydration sequences in negatives, impacting training results.

We used t-distributed stochastic neighbor embedding (t-SNE) to visualize differences between original negative samples and those refined by confidence learning. Figure 3C shows that refined negative samples are more clustered compared to original negatives, indicating effective noise removal and reliable negative sample selection through confidence learning. It confirms the efficacy of the confidence learning algorithm in screening negative samples.

We used the same number of samples for t-SNE visualization before and after confidence learning, as shown in Supplementary Fig. S6. It can be observed that the negative samples are scattered around the edges, while the samples after confidence learning exhibit clustering at the center. This demonstrates that confidence learning is effective in sample selection and contributes to more efficient optimization of negative samples.

### 3.7 Gene ontology and KEGG analysis of S-sulfhydrated proteins

We statistically analyze the enriched biological processes, cell component and molecular functions with the gene ontology annotations with Fisher-exact test for S-sulfhydrated proteins, and map all the S-sulfhydrated proteins to the Kyoto Encyclopedia of Genes and Genomes (KEGG) pathways to further explore functional aspects of S-sulfhydration substrates. The statistical results are shown in Fig. 3D and E.

We can conclude from Fig. 3D that S-sulfhydration prefers to occur at various cellular level process, such as cell communication. Besides, S-sulfhydrated proteins are active at the extracellular region and vesicle-related region. S-sulfhydration also involves in various binding activity between proteins, various ligands, and compounds. Taken together, these observations show that S-sulfhydration plays an indispensable role in human body.

We can detect from Fig. 3E that sulfhydration modified proteins are related to multiple diseases, especially neurodegenerative diseases, including Parkinson disease, Prion disease, Amyotrophic lateral sclerosis, Huntington disease, and Alzheimer disease. These results concluded demonstrate that the study of sulfhydration mechanism contributes to the understanding of disease and pharmaceutical industry.

Detailed analyses are narrated in Supplementary Materials.

### 3.8 Comparison with pCysMod

The purpose of Sul-BertGRU is to accurately predict S-sulfenylation sites. Here, we compared the performance of Sul-BertGRU and pCysMod on an independent test set. Table 3 presents the comparison results of the two methods. It can be observed that Sul-BertGRU outperforms pCysMod in most metrics, such as Sn, ACC, and MCC, with a significantly higher Sn. Overall, Sul-BertGRU demonstrates superior performance in predicting S-sulfenylation sites.

**Table 3.** Performance results of pCysMod and Sul-BertGRU.^a^

Methods	Sn	Sp	ACC	MCC
pCysMod	0.7190	0.7773	0.7545	0.5082
Sul-BertGRU	**0.8582**	**0.6824**	**0.7744**	**0.5513**

a The bold font indicates the performance values for Sul-BertGRU.

### 3.9 Comparison of performance with different sequence window lengths

To explore the impact of sequence window size on prediction performance, we conducted experiments using sequence windows of Cys ± 15, Cys ± 10, and Cys ± 5. The results show that Cys ± 15 performs the best, followed by Cys ± 10, and then Cys ± 5. Although the performance of Cys ± 10 and Cys ± 5 is not significantly different from that of Cys ± 15, the latter achieves the best performance. This indicates that while sequences near the central cysteine contain rich information, sequences at the edges also carry critical information that cannot be ignored. The experimental results are shown in Supplementary Fig. S7.

## 4 Conclusion and discussion

To advance cell biology and understand diseases, we developed Sul-BertGRU, an S-sulfhydration site prediction model using a multi-directional GRU and an entropy-enhanced BERT framework. The model segments the protein sequence into left, right, and whole sequences for GRU-based feature extraction and BERT preprocessing. Features are then merged and refined by attention, followed by local feature recognition using CNN. Validation experiments show significant results, though direct comparisons are not possible due to the unique dataset. Future work will incorporate additional structural information to improve feature extraction and prediction accuracy.

## Supplementary Material

btaf078_Supplementary_Data

## Data Availability

All data used in this study are available at https://github.com/Severus0902/Sul-BertGRU/.
