# Supplementary material for: Sul-BertGRU: an ensemble deep learning method integrating information entropy-enhanced BERT and directional multi-GRU for S-sulfhydration sites prediction
Source: Bioinformatics. 2025 Feb 20;41(3):btaf078. doi: 10.1093/bioinformatics/btaf078 (PMC11908646; doi:10.1093/bioinformatics/btaf078)
Supplement: btaf078_Supplementary_Data [file btaf078_supplementary_data.pdf]

Supplementary Materials for Sul-BertGRU: An Ensemble Deep Learning Method integrating Information Entropy-enhanced BERT and Directional Multi-GRU for S-sulfhydration Sites Prediction

1 Directional Details of Protein Sequences

In this section, we will provide a detailed description of the different directional combinations of protein sequences.

Considering the potential directionality of enzymatic reactions, the three GRU models can process three-part protein sequences in various orientations, resulting in eight possible choices:

(1) Both halves and the full sequence are processed from left to right (LLL).

(2) Left half from left to right, right half from right to left, full sequence from left to right (LRL).

(3) Both halves from left to right, with the full sequence from right to left (LLR).

(4) Left half from left to right, right half from right to left, and the full sequence also from right to left (LRR).

(5) All sequences, including both halves and the full, from right to left (RRR).

(6) Left half from right to left, right half from left to right, and the full sequence from right to left (RLR).

(7) Left half and right half from right to left, with the full sequence from left to right (RRL).

(8) Left half from right to left, right half from left to right, and the full sequence from left to right (RLL).

After extracting directional features from the three parts of the protein sequence, we input these features into the attention module for processing. Following this, the outputs from both the GRU and attention modules are extracted independently, concatenated, and then input into the CNN module. This process allows for a thorough capture of local features that might be missed otherwise.

2 Multi-head self-attention Implementation Details

To capture long-term dependencies in protein sequences, we adopt the self-attention mechanism, widely recognized for its robust capability in various tasks such as visual recognition, image classification, and disease prediction. The feature information of the three parts of the protein sequences is extracted, and further processed using a multi-head self-attention module.

Initially, we compute the query matrix Q, key matrix K and value matrix V as follows:

$$Q = M_t W^Q, K = M_t W^K, V = M_t W^V \tag{1}$$

where M_t is the GRU output, W^Q, W^K and W^V are trainable matrices.

The attention matrix A' is computed as:

$$A' = softmax(\frac{QK^\top}{\sqrt{d_k}}) \tag{2}$$

where $\sqrt{d_k}$is the dimension of K.

In the multi-head mechanism, each head's attention is given by:

$$head_i = Attention(Q_i, K_i, V_i) = A'_i V_i, i = 1, 2, ..., n \tag{3}$$

The final output matrix A is obtained by concatenating the results from all heads:

$$A = Concact(head_1, \ldots, head_n)W^O \quad (4)$$

where W^O is a learnable matrix.

3 CNN Implementation Details

In this section, we will introduce the implementation details of the CNN module. In the process of feature extraction from protein sequences, the GRU module and the self-attention mechanism primarily focus on sequence-wide dependencies. This focus might cause them to miss local details within some segments of the sequences. Therefore, we input the feature matrices from the GRU module and the attention module into the CNN to locally sense and fuse information, thereby obtaining richer local feature information between each amino acid residue. The CNN comprises three convolutional layers and three pooling layers.

- **Convolutional layer:** The convolutional layer processes the feature matrices A and M_t, which are derived from the attention mechanism and the GRU module, respectively. This step enhances the extraction of local features, thereby enriching the overall feature information captured. After obtaining the local features, we use *ReLU* function to nonlinearly map the output, which is calculated as follows:

$$Z^{(k)}{}_1 = \text{ReLU}(A \times C^{(k)} + a^{(k)}), \qquad k = 1, 2, 3 \quad (5)$$

$$Z^{(k)}{}_2 = \text{ReLU}(M_t \times C^{(k)} + a^{(k)}), \qquad k = 1, 2, 3 \quad (6)$$

  where, k denotes the number of convolutional layers, which is set to a maximum of 3 in this paper, C denotes the size of the convolutional kernel for each convolutional layer, which is set to 2 in our model, and a denotes the bias term, which is enabled by default in our model.

- **Pooling layer:** The pooling layer reduces the size of the input features, preserving essential information and eliminating redundancy. To enhance the extraction of local features, we utilize the output matrix Z from the convolutional layer as the input for the pooling layer, applying an average pooling operation. This involves averaging all elements within the feature matrix and forwarding the result to the subsequent layer. This approach ensures that all features within the matrix are considered. The computational steps for each of the three pooling layers are summarized and simplified as follows:

$$Y^{(k)}{}_1 = \frac{1}{|Z^{(k)}{}_1|} \sum_{(p,q) \in Z^{(k)}{}_1} e_{kpq}, k = 1, 2, 3 \quad (7)$$

$$Y^{(k)}{}_2 = \frac{1}{|Z^{(k)}{}_2|} \sum_{(p,q) \in Z^{(k)}{}_2} e_{kpq}, k = 1, 2, 3 \quad (8)$$

  where $|Z^{(k)}{}_1|$ and $|Z^{(k)}{}_2|$ represent the total number of elements in the feature matrix for each k, ande_{kpq} is the specific element located at position (p, q) within the $k$feature matrix region.

  Eventually, the two parts of the matrix undergo further feature extraction by the CNN. Subsequently, the outputs from the final pooling layer are concatenated to form the ultimate feature matrix Y, which is then input into a fully connected network for subsequent classification and prediction tasks:

$$Y = Concact(Y_1^{(3)}, Y_2^{(3)}) \quad (9)$$

4 Additional hyperparameters analysis

4.1 hyperparameters in GRU

For the GRU model, increasing the dimension of the hidden layers is important for improving the model's ability to learn richer and more complex feature information, thus enabling better handling

of long protein sequences. Additionally, the number of layers in the hidden layer is also crucial, as a greater number of layers allows for more nonlinear transformations to be applied to the protein sequences, thereby facilitating the extraction of more complex features and long-term dependencies. In this study, we set the dimensions of the hidden layers to 64, 128, 256, and 512, and the number of layers of the hidden layers to 1, 2, 3, and 4, respectively. During the training of the model, all other parameters remain unchanged, maintaining a consistent batch size and learning rate. Additionally, the random seed is set to 42. To assess the predictive performance of the model on the test set, Acc and MCC are selected as the evaluation metrics.

Upon evaluating the predictive performance of models with various parameter configurations as depicted in **Figure 2(A)**, the optimal results are achieved when the model is configured with three GRU hidden layers and a dimension size of 128. This indicates that the GRU effectively learns sequence features at a parameter combination of 3,128 and is effective in identifying S-sulfhydrylation sites.

4.2 hyperparameters in CNN

In CNN, the performance is significantly influenced by the size of the convolutional kernels and the number of convolutional layers. To explore how the size of convolutional kernels and the number of layers in a CNN impact the model's predictive accuracy, a series of validation experiments are performed. We configure the convolutional kernel sizes at 1, 2, 3, and 4, and adjust the number of CNN layers to 1, 2, and 3 for our experiments. We choose Acc and MCC on the test set as the evaluation criterion. Apart from the convolutional kernel size and the number of CNN layers, all other parameters within the model remained constant. As shown in **Figure 2(A)**, optimal results on the test set, with the highest Acc and MCC, are observed when the convolutional kernel size is set at 2 and the number of CNN layers is 3. Additionally, the model's Acc improves as the number of CNN layers increases. This is because more layers can extract more local features from the protein sequence, while fewer layers may not be able to fully capture its features. Thus, enhancing the number of CNN layers contributes effectively to better prediction performance of the model.

4.3 The number of heads of the multi-head self-attention module

For the multi-head self-attention mechanism, selecting the optimal number of attention heads is crucial for enhancing the extraction and representation of protein sequence features. In this study, we carry out a series of experiments to explore how varying the number of self-attention heads impacts their effectiveness. Given that an excessive number of self-attention heads can overly complicate the prediction model and increase parameter counts, we opt for self-attention mechanisms with 1, 2, 4, and 8 heads for our study. Apart from the number of heads in the self-attention mechanism, all other model parameters remain unchanged. The outcomes of this configuration are displayed in **Figure 2(B)**.

The findings indicate that the GRU model is crucial in identifying the directionality of S-sulfhydrylation sites within the Sul-BertGRU framework. The results demonstrate that the model equipped with 4 heads outperforms those with different numbers of heads during the prediction process. As the number of self-attention heads increases, the model's performance exhibits variability. This may occur because additional self-attention heads facilitate the extraction of more diverse feature information from both upstream and downstream segments of the sequence, thereby enhancing the model's ability to learn from protein sequences. Nonetheless, an excessive number of self-attention heads can lead to increased complexity within the model, potentially diminishing its predictive performance. Hence, setting the number of self-attention mechanism heads to 4 proves effective for our model in accurately identifying and predicting S-sulfhydrylation sites.

5 Extended Analysis of the GRU Model

In Sul-BertGRU, we employ a GRU to extract directional features of protein sequences. LSTM is known for its strong memory and long-term dependency-capturing ability, and is also suitable for processing long sequence data of proteins. To assess the GRU's capability in extracting directional features, we conduct a comparative analysis with the LSTM model. Additionally, since the GRU model can only make predictions based on the upstream information of the protein sequence, while BiGRU and BiLSTM are able to capture both the upstream and downstream information of the

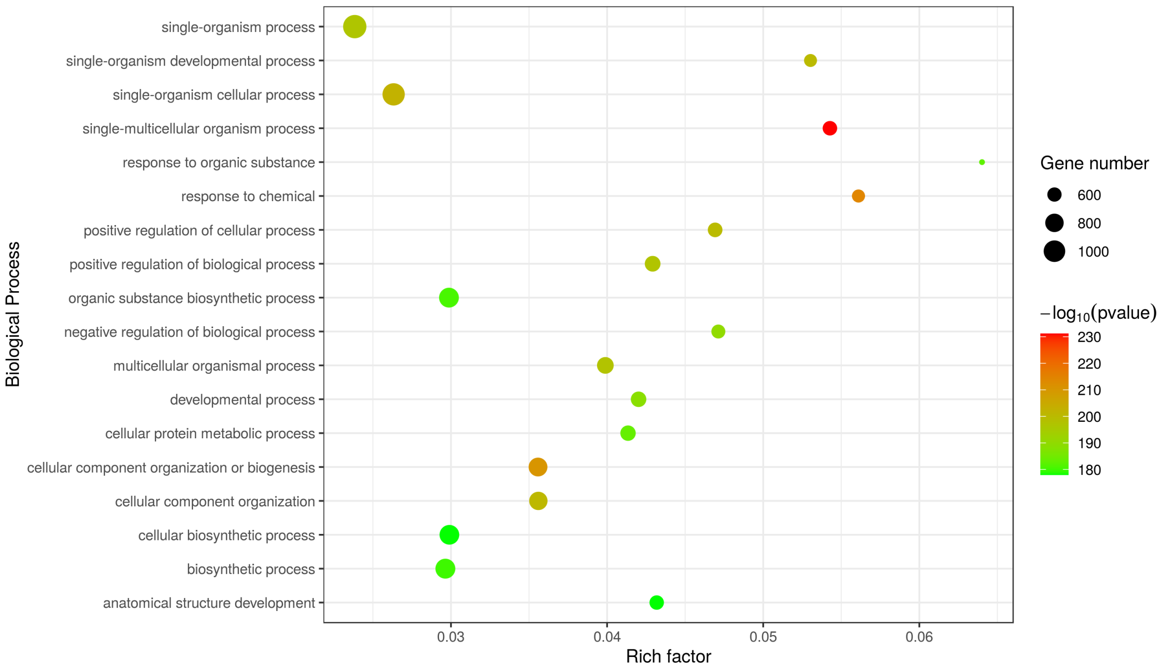

Figure S1: The top statistically over-represented terms of biological processes ($p - value < 0.01$) related to S-sulfhydration modified proteins.

sequence, to further explore whether the formation of S-sulfhydrylation sites is influenced by the sequence's upstream and downstream information, we conduct additional comparisons between BiGRU and BiLSTM. The findings are illustrated in **Figure 2(C)**.

In the test of extracting directional features of protein sequences, the GRU model performed the best among the four models. Compared with those of the LSTM model, its Sn, Sp, Pre, Acc, MCC, and AUC are 1.31%, 2.46%, 1.82%, 3.72%, and 1.88% greater, respectively. This could be attributed to the GRU's fewer parameters, which lessen the likelihood of overfitting during training, thereby enhancing its ability to capture sequence feature information more effectively. Additionally, in the comparison with BiGRU and BiLSTM, although the BiLSTM model is slightly better than our model, all other metrics are reduced to different degrees. When the sequence information is aggregated, BiGRU and BiLSTM are bidirectional, while the direction of the GRU is fixed, which implies that there is a fixed direction for extracting the S-sulfhydrylation modification sites.

6 Gene ontology analysis of S-sulfhydrated proteins

We statistically analyze the enriched biological processes, cell component and molecular functions with the gene ontology (GO) annotations with Fisher-exact test for S-sulfhydrated proteins. The statistical results are shown in **Figure S1**, **Figure S2** and **Figure S3**.

We can clearly see from **Figure S1** that S-sulfhydration prefers to occur at various cellular level process, including single-organism cellular process (GO:0044763), cellular component organization (GO:0016043), positive regulation of cellular process (GO:0048522) and multicellular organismal process (GO:0032501). Besides, it involves multiple chemical reactions and pathways, such as cellular macromolecule metabolic process (GO:0044260), cellular protein metabolic process (GO:0044267), organic substance biosynthetic process (GO:1901576), biosynthetic process (GO:0009058) and cellular biosynthetic process (GO:0044249). Although the number of genes matched by the response to organic substance (GO:0010033) is small, the rich factor is large. Response to organic substance means Any process that results in a change in state or activity of a cell or an organism (in terms of movement, secretion, enzyme production, gene expression, etc.) as a result of an organic substance stimulus.

As for cell component in **Figure S2**, among the top statistically over-represented terms related to

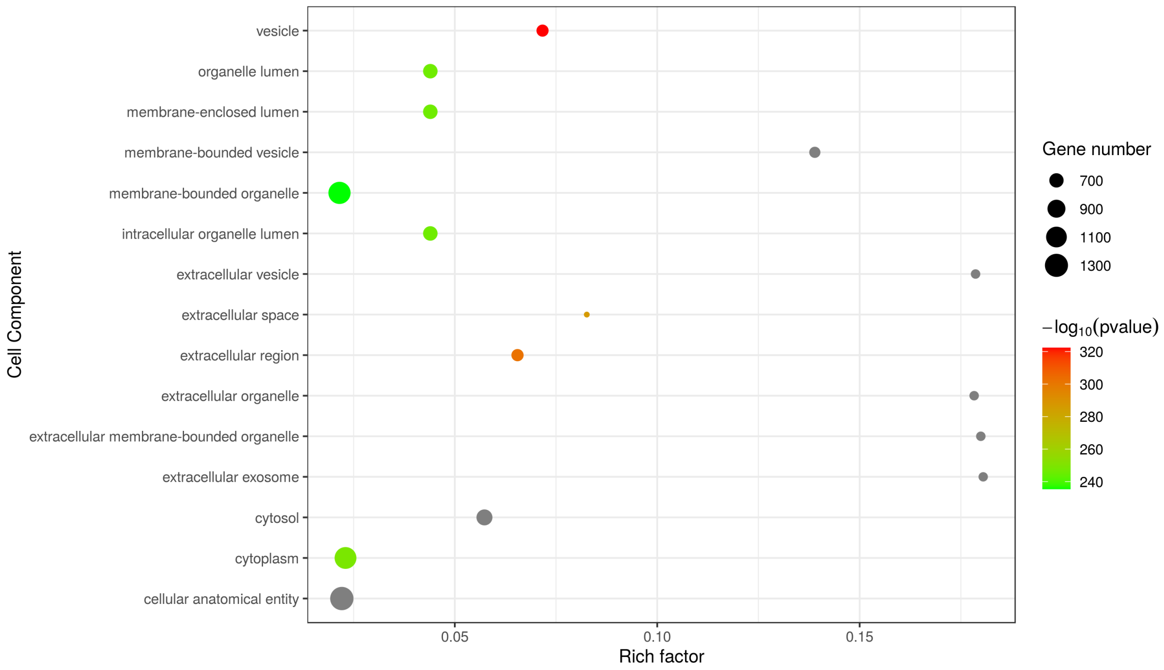

Figure S2: The top statistically over-represented terms of cell component ($p - value < 0.01$) related to S-sulfhydration modified proteins.

S-sulfhydration modified proteins, six are different extra cellular positions including extracellular organelle (GO:0043230), extracellular vesicle (GO:1903561), extracellular membrane-bounded organelle (GO:0065010), extracellular exosome (GO:0070062), extracellular region (GO:0005576) and extracellular space (GO:0005615). It indicates that S-sulfhydration modified proteins are active out of cellular. The components associated with cell membranes are also highly enriched, including membrane-bounded vesicle (GO:0031988), extracellular membrane-bounded organelle (GO:0065010), membrane-enclosed lumen (GO:0031974) and membrane-bounded organelle (GO:0043227). [1] suggests that exogenous H2S sulfhydrates K(V)4.3 to decrease the membrane potential, thereby enhancing the basal tension of gastric antral smooth muscle. Therefore, we can conclude that S-sulfhydration plays an active role in cell membrane and is associated with some changes in organism. Besides, there are several organelle-related cell component (extracellular organelle (GO:0043230), extracellular membrane-bounded organelle (GO:0065010), organelle lumen (GO:0043233) and membrane-bounded organelle (GO:0043227)) and vesicle-related cell component (extracellular vesicle (GO:1903561),membrane-bounded vesicle (GO:0031988) and vesicle (GO:0031982)) among the top statistically over-represented terms. Furthermore, cytosol (GO:0005829), cytoplasm (GO:0005737) and cellular anatomical entity (GO:0110165) are also enrich, which means that S-sulfhydrated proteins are active no matter in cellular or out of cellular.

For molecular function in **Figure S3**,, we can obviously find that all the top statistically over-represented terms are about binding. We can infer from it that S-sulfhydration may promote binding between proteins, various ligands and compounds which may cause a lot of diseases.

Taken together, these observations show that S-sulfhydration plays an indispensable role in human body.

7 KEGG analysis of S-sulfhydrated proteins

We map all the S-sulfhydrated protein used in our study to the Kyoto Encyclopedia of Genes and Genomes (KEGG) pathways to further explore functional aspects of S-sulfhydration substrates. The top 10 enriched pathways are listed in Fig 3(E) and the statistical result of significant pathways is shown in **Figure S4**, ($p - value < 0.01$).

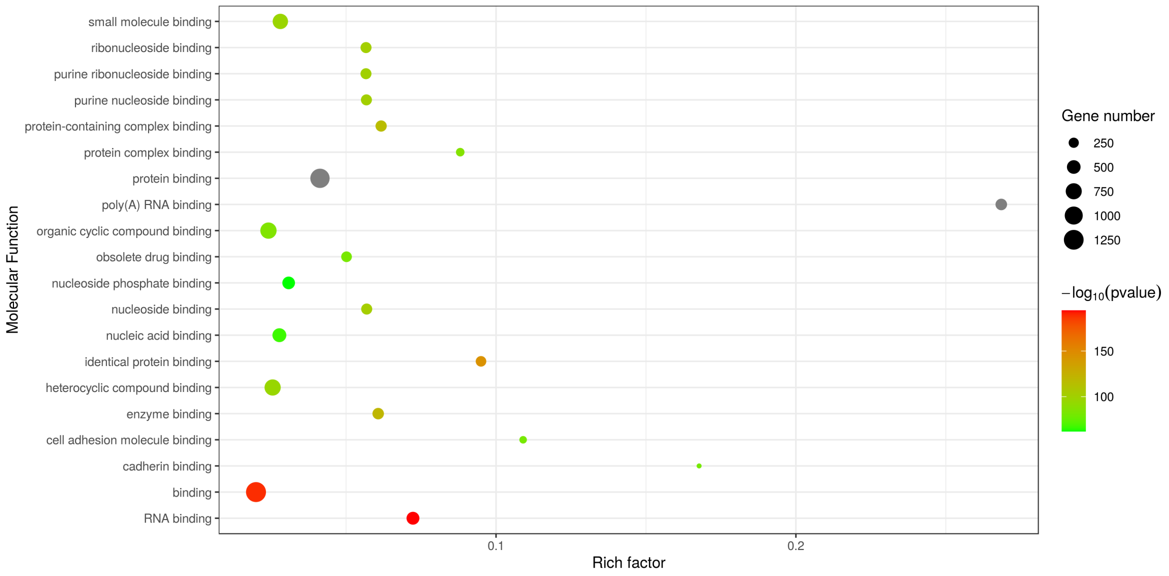

Figure S3: The top statistically over-represented terms of molecular function ($p-value < 0.01$) related to S-sulfhydration modified proteins.

We can detect from **Fig 3(E)** that S-sulfhydration modified proteins are related to multiple diseases, especially neurodegenerative diseases, including Parkinson disease, Prion disease, Amyotrophic lateral sclerosis, Huntington disease and Alzheimer disease. These results concluded demonstrate that the study of sulfhydration mechanism contributes to the understanding of disease and pharmaceutical industry. Besides, we can clearly find that metabolism occupy a large proportion in **Figure S4**. Among metabolism-related terms, carbohydrate metabolism is the most significant one. Abnormalities in carbohydrate metabolism involves in various diseases, such as brain disease [2], kidney disease [3] and celiac disease [4], which is consistent with the results shown in **Fig 3(E)**. Except metabolism pathway, a large portion of S-sulfhydrated proteins are related to genetic information processing, cellular processes and organismal systems, which is consistent with results of gene ontology analysis.

References

[1] Miao Yu, Haining Du, Bingzhu Wang, Jian Chen, Fangping Lu, Shuo Peng, Yu Sun, Ning Liu, Xiaojiao Sun, Shiyun Dong, Yajun Zhao, Yan Wang, Dechao Zhao, Fanghao Lu, and Weihua Zhang. Exogenous h¡sub¿2¡/sub¿s induces hrd1 s-sulfhydration and prevents cd36 translocation via vamp3 ubiquitylation in diabetic hearts. *AGING AND DISEASE*, 11(2):286–300, APR 2020.

[2] G. M. Perrin, ALTSCHULE MD, P. D. Holliday, and R. M. Goncz. Carbohydrate metabolism in brain disease. xii. effects of epinephrine on intermediary carbohydrate metabolism in normal and schizophrenic subjects. *A.m.a.archives of Internal Medicine*, 103(5):730, 1959.

[3] Eberhard Ritz, Marcin Adamczak, and Andrzej Wiecek. Carbohydrate metabolism in kidney disease and kidney failure. *Nutritional Management of Renal Disease (Third Edition)*, pages 17–30, 2013.

[4] Ensieh. Khalkhal, Mostafa. Rezaei-Tavirani, Mohammadreza. Razzaghi, Sina. Rezaei-Tavirani, Hakimeh. Zali, and Mohammad. Rostamii-Nejad. The critical role of dysregulation of antioxidant activity and carbohydrate metabolism in celiac disease. *Gastroenterology and hepatology from bed to bench*, 12(4):340–347, 2019.

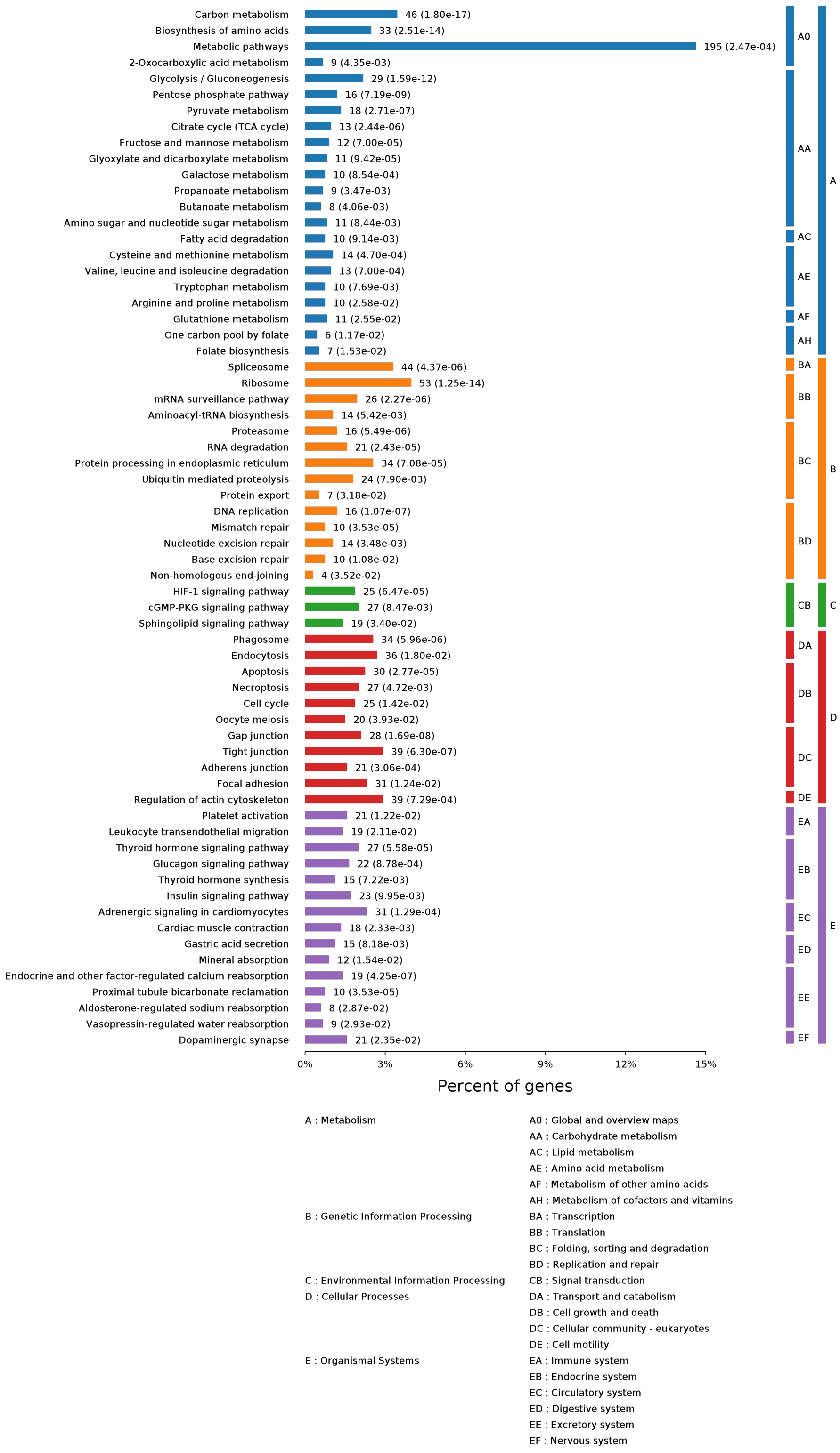

Figure S4: The statistical result of significant pathways$p - value < 0.01$).

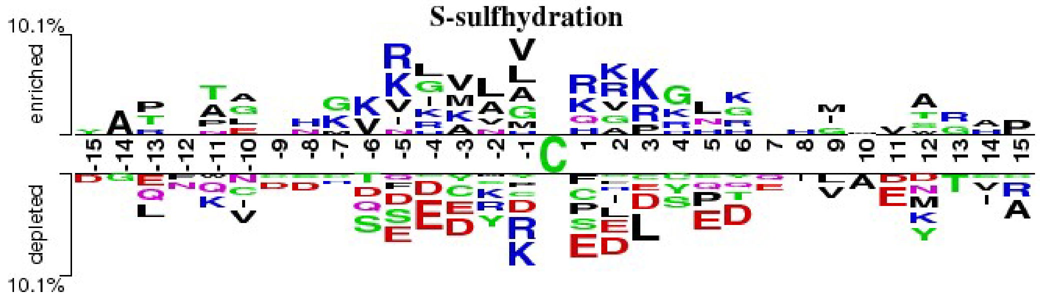

Figure S5: Two sample logos of the compositional biases around S-sulfhydration sites without K/R residues compared with nonsulfhydration sites.

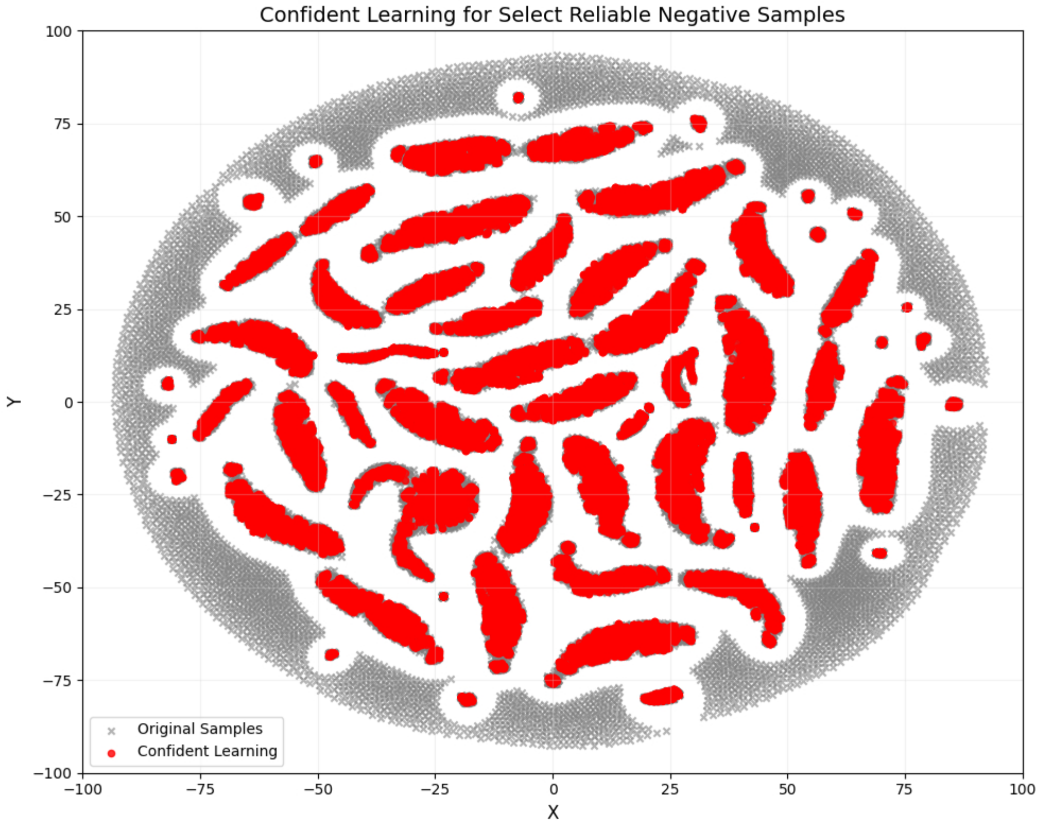

Figure S6: t-SNE results of confident learning for selecting reliable negative samples (1:1).

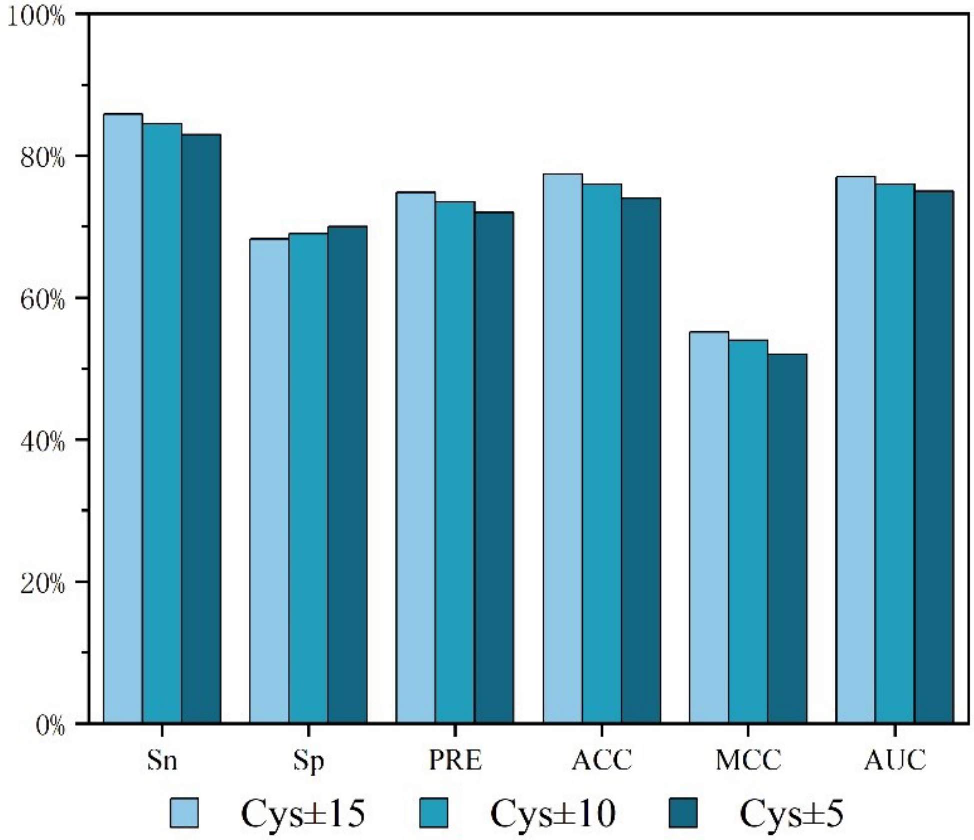

Figure S7: Performance results for different sequence window sizes.
